# Stealth and deception: Adaptive motion camouflage in hunting broadclub cuttlefish

**DOI:** 10.1126/sciadv.adr3686

**Published:** 2025-03-26

**Authors:** Matteo Santon, Jolyon Troscianko, Charlie D. Heatubun, Martin J. How

**Affiliations:** ^1^ School of Biological Sciences, University of Bristol, Bristol, UK.; ^2^ Centre for Ecology and Conservation, University of Exeter, Penryn, UK.; ^3^ Faculty of Forestry, University of Papua, Manokwari, Indonesia.; ^4^ Regional Research and Innovation Agency (BRIDA), Manokwari, Indonesia.

## Abstract

Maintaining camouflage while moving is a challenge faced by many predators. Some exploit background motion to hide while hunting, and others may use coloration and behavior to generate motion noise that impairs detection or recognition. Here, we uncover a unique form of motion camouflage, showing that broadclub cuttlefish pass dark stripes downward across their head and arms to disguise their hunting maneuvers. This “passing-stripe” display reduces the probability of response to predatory expanding stimuli by prey crabs in a lab-based experiment, is modulated according to approach speed during a hunt, and generates a motion pattern that is different from that of looming predators. This form of motion camouflage likely functions by overwhelming the threatening motion of the approaching predator with nonthreatening downward motion generated by the rhythmic stripes.

## Teaser

Wild broadclub cuttlefish deceive prey with a hunting display that camouflages their approach with downward moving stripes.

## INTRODUCTION

Camouflage is disrupted by motion (*1*–*3*). Yet, all predators must hunt to survive, which often produces strong motion signals that make them highly conspicuous. Natural scenes are also not still. Background motion can be generated by physical forces such as winds or currents displacing vegetation, by dynamic illumination such as the caustic flicker that plays across shallow water benthos, but also by egocentric movement that generates background optic flow (*4*, *5*). Some predators can make use of this background motion to camouflage their own movement cues. Stalking hoverflies, dragonflies, and falcons can fly in such a way as to match the optic flow pattern in their moving prey’s visual system (*6*–*9*), and some arboreal animals such as vine snakes and stick insects mimic the swaying movement of wind-blown vegetation while locomoting [(*10*, *11*) but see (*12*)]. Other animals may instead camouflage by generating motion noise and thus impair the detection, recognition, or interpretation of their movements. Proposed strategies are protean movement, i.e., moving in an unpredictable way (*13*), motion confusion (also known as dazzle), by which high-contrast markings can alter the estimation of trajectory and speed of a target (*2*, *14*–*17*), dynamic color change (*3*, *18*, *19*), and flicker-fusion camouflage, which occurs when the target moves at speeds that make its pattern appear to blur, thus becoming less apparent against the background (*20*). Evidence for camouflage in motion in the wild is scarce (*3*), and few studies have investigated the effectiveness of generating motion noise as a camouflage strategy in a real biological interaction.

### Dynamic skin patterns in cuttlefish

Cuttlefish can dynamically switch among different skin patterns via fine neural control of chromatophores (*21*–*24*). Because of their unique ability to modulate such skin patterns according to specific behavioral tasks, cuttlefish are an ideal system to investigate camouflage in motion. For example, the European cuttlefish *Sepia officinalis* rapidly switches to low contrast gray patterns when moving across different substrates and adjusts body reflectance according to natural backgrounds (*25*, *26*). Instead of dynamically matching the background, one cuttlefish species may have adopted a very different strategy to camouflage its movement while stalking prey, the broadclub cuttlefish *Sepia latimanus*.

### The broadclub cuttlefish has a unique hunting technique

**Fig. 1.**
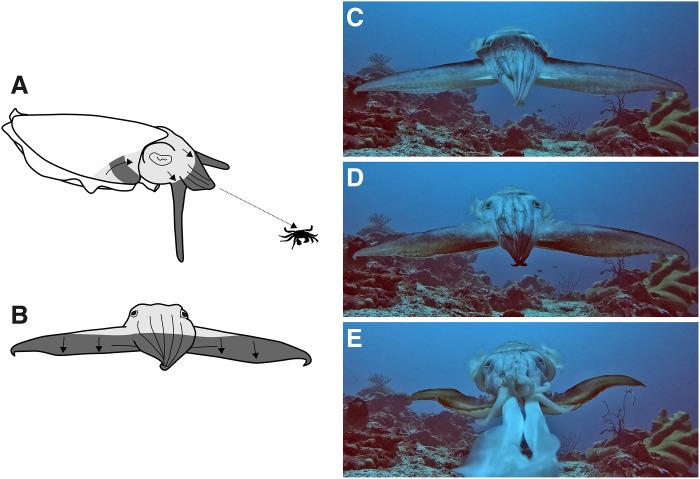
The hunting display of *S. latimanus*. (**A**) Dorso-lateral view of the broadclub cuttlefish passing dark stripes while hunting prey crab. (**B**) View of the display from the perspective of the hunted crab. (**C** to **E**) Sequential video frames of broadclub cuttlefish hunting a crab from prey visual perspective. Scheme: M.J.H., pictures: M.S.

While approaching crab prey, this cuttlefish species changes the appearance of its head to a homogeneous white color, stretches six of its arms forward into a tight cone and the remaining two arms are stretched laterally with their broad flat surfaces pointing forward. Then, the cuttlefish passes highly contrasting dark stripes in a downward direction across the head and arms (Fig. 1, A to D, and movies S1 and S2) until it strikes its prey (Fig. 1E) (*27*). This has been popularly described in natural history documentary films as a “mesmerizing” display and is considered to somehow provide the cuttlefish with a hunting advantage during the final moments of attack. In the context of this hunting display, the hunted crab views the approaching cuttlefish through a relatively low-resolution visual system that is heavily reliant on motion estimates for guiding behavior (*28*, *29*). In particular, the detection of looming stimuli by crabs is known to be optimized for motion cues generated by approaching predators (*30*, *31*). In this study, we investigated whether the broadclub cuttlefish uses the passing-stripe display to camouflage its approach to crab prey.

## RESULTS

### Effect of passing stripe display on prey response

**Fig. 2.**
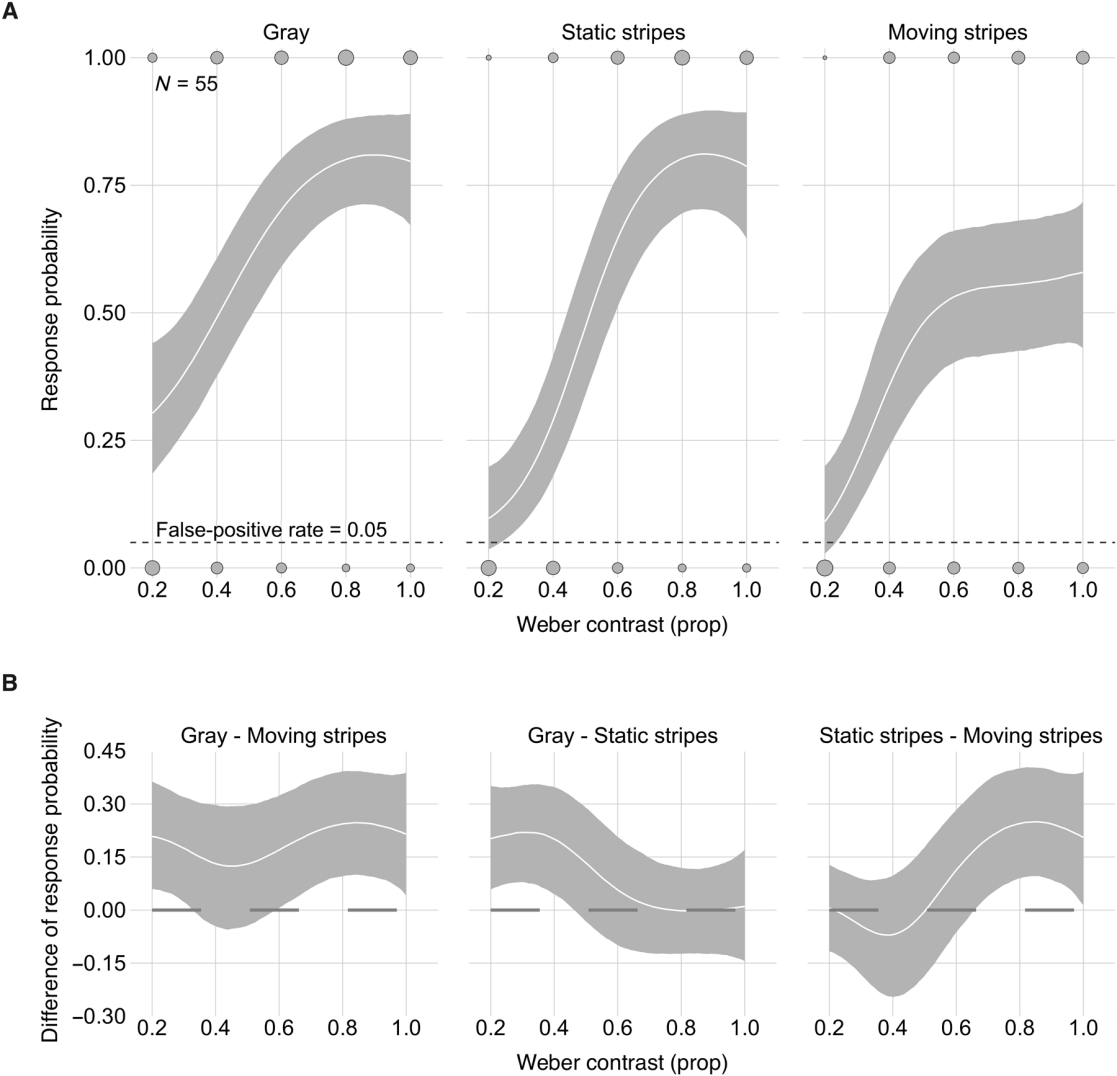
Expanding stimuli with downward moving stripes are less likely to elicit a response in prey crabs. (**A**) Model posterior predictions of median response probabilities (solid lines) and their 95% CIs (shaded areas) as a function of stimulus contrast with the background. Raw data counts are mapped to point area (gray filled circles), i.e., the larger the circle, the more observations overlap. (**B**) Comparisons between stimuli as a function of WC. Solid lines represent median differences, shaded areas their 95% CIs.

By tethering shore crabs *Carcinus maenas* over a Styrofoam treadmill in front of a liquid crystal display (LCD) monitor (*32*), we tested whether an expanding predator stimulus with moving stripes—a proxy for the approaching cuttlefish—elicits a weaker response compared to control stimuli in prey that are naïve to passing stripe hunting displays. Crabs were alternately presented with three elliptical expanding stimuli without stripes, with stationary stripes, or with downward moving stripes. Stimuli were presented in a range of Weber contrasts (WC) against a homogeneous gray background. Crabs were more likely to react to high-contrast stimuli (e.g., WC = 1) without stripes or with stationary stripes than with stripes moving in a downward direction (Fig. 2, A and B, and table S1). These results suggest that the passing stripe display could be an effective strategy to increase predation success when the broadclub cuttlefish is hunting crabs.

### Cuttlefish approach speed influences stripe temporal frequency

**Fig. 3.**
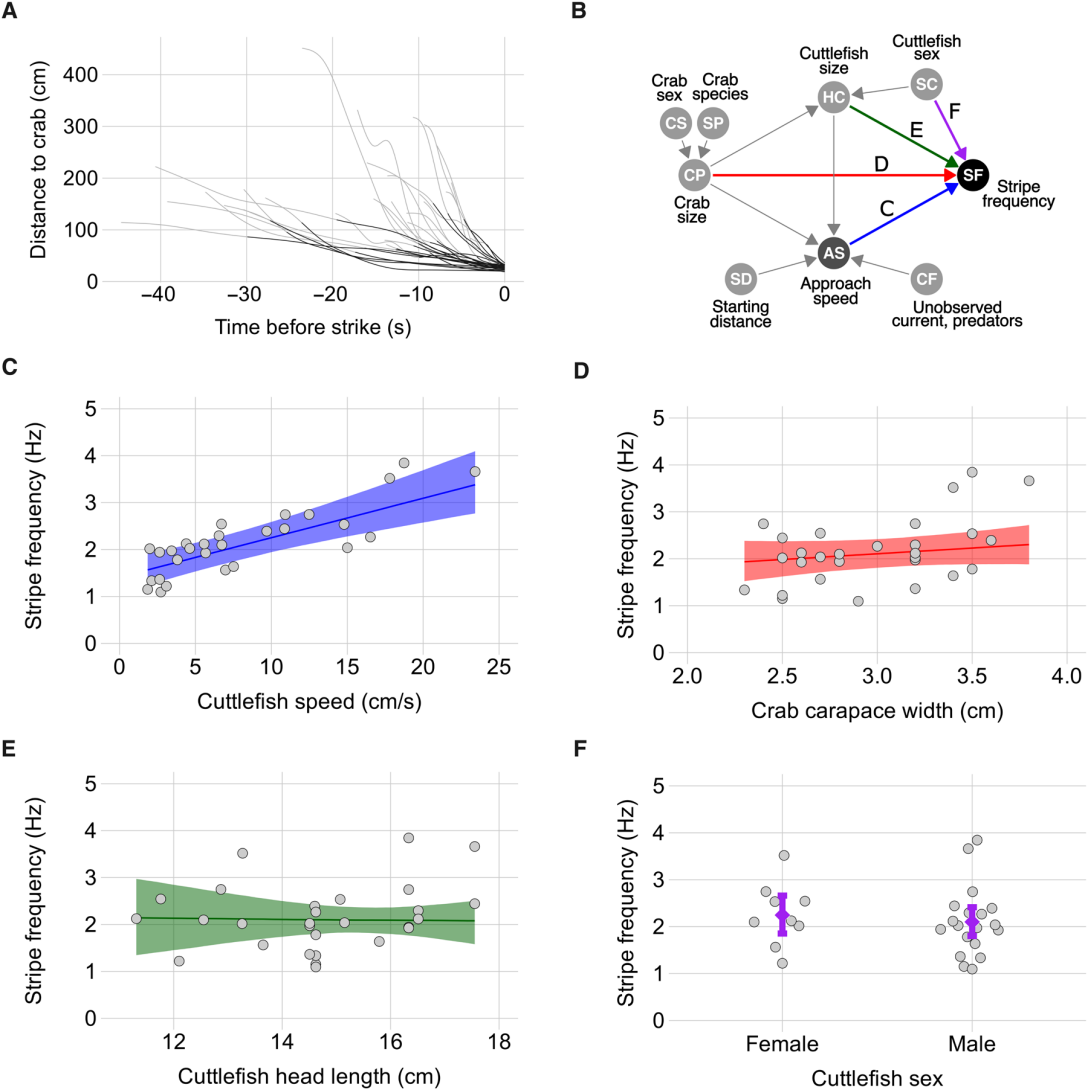
Broadclub cuttlefish tunes stripe temporal frequency based on the speed of its hunting approach. (**A**) Distance of cuttlefish from prey crabs as a function of time before the strike. Each of the 28 lines represents one hunting event. The lines become darker when the cuttlefish starts using the stripe display. (**B**) DAG showing the relationship between observed and unobserved variables. Arrows indicating direct effects of investigated variables on the response “stripe frequency” are color coded according to the subpanels below. (**C**) Model posterior predicted median stripe temporal frequency (solid line) and its 95% CIs (shaded area) as a function of cuttlefish approach speed. Filled circles (*N* = 28) are raw data for each hunting event from 17 cuttlefish. (**D** to **F**) The same for the other predictors of interest.

We then filmed cuttlefish hunting with the passing stripe display in the field using a multicamera rig (movie S1). We used these videos to reconstruct hunting trajectories in three dimensions and estimate metrics to describe the natural variability of this hunting display. We recorded 28 passing stripe hunting displays from at least 17 different individuals (9 females and 8 males, with average mantle length of 22.1 ± 3.2 and 28.6 ± 4.6 cm respectively). After spotting prey, the broadclub cuttlefish followed a two-phase approach: First, the cuttlefish performed a rapid approach over the first 58 ± 18% of the distance range; then, for the final close-range approach, the cuttlefish switched to the passing stripe display (Fig. 3A). The initial rapid approach was, on average, 10 ± 13 cm/s faster than the rest of the hunting trajectory with the passing stripe display, during which the cuttlefish approached at a slower average speed of 8.2 ± 6 cm/s. In all hunting events, moving stripes were only used during the final phase of the attack when the predator was closer to prey, from an average distance of 97.8 ± 32.3 cm (Fig. 3A). The average temporal frequency of stripes moving down the head and arms of the cuttlefish was 2.2 ± 0.7 Hz.

To investigate whether the speed of the final phase of the cuttlefish approach directly influenced the temporal frequency of stripe expression, we used a causal framework (*33*) based on the directed acyclic graph (DAG) of Fig. 3B to infer, with a robust linear regression, the causal effect that the approach speed of the cuttlefish has on stripe temporal frequency while adjusting for size of prey, size, and sex of the cuttlefish. Approach speed when displaying the stripes influenced stripe frequency: For a one SD increase in approach speed (5.9 cm/s), the frequency of the stripes increased by 0.50 Hz [95% confidence intervals (CIs): 0.31, 0.72] (Fig. 3C). With the same model, we also estimated the direct causal effect of the size of prey crabs on stripe frequency and found very little influence: For a one SD increase in crab carapace width (0.4 cm), stripe frequency increased by 0.1 Hz (95% CIs: −0.07, 0.26) (Fig. 3D). Cuttlefish size and sex also had negligible influence on stripe frequency: For a one SD increase in head length (1.7 cm), stripe frequency decreased by 0.02 Hz (95% CIs: −0.33, 0.28) (Fig. 3E); and compared to an equally large female hunting the same sized prey at identical approach speed, a male’s stripe frequency would only be lower by 0.14 Hz (95% CIs: −0.73, 0.44) (Fig. 3F). The effects of prey size, cuttlefish size, and sex were almost entirely mediated by the speed of approach of the cuttlefish (table S2). These results suggest a clear link between the saliency of the approaching cuttlefish cue and the strength of the motion generated by moving stripes.

### The stripe display generates unusual motion patterns for approaching predators

**Fig. 4.**
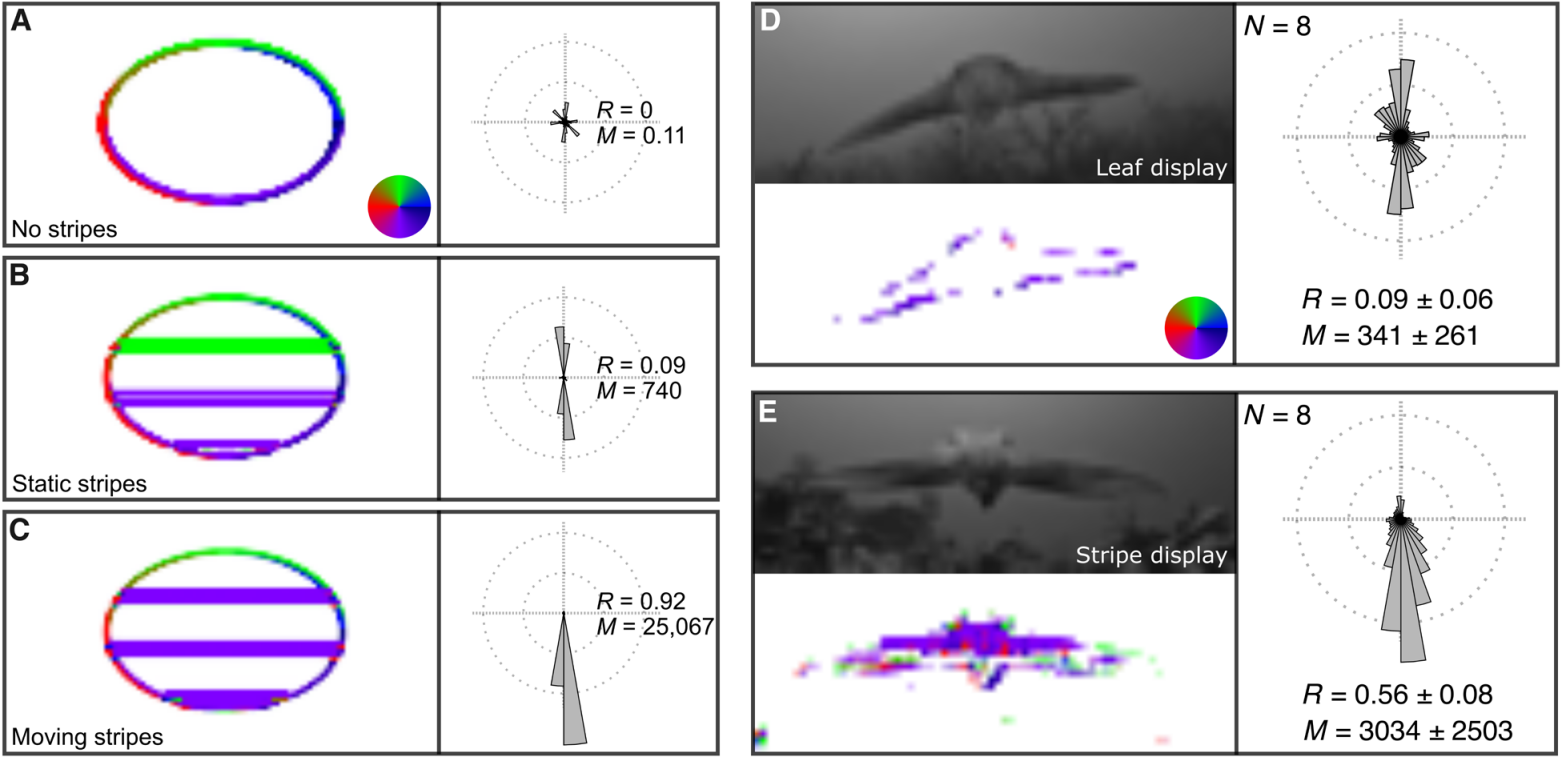
Motion patterns generated by the stimuli of the treadmill experiment and sequences of broadclub cuttlefish hunting crab. (**A** to **C**) Motion displacement (left) of the fully expanding stimuli used in the treadmill experiment. (**D** and **E**) Frames of hunting sequences spatiotemporally sampled to crab vision (top-left) with their motion displacement (bottom-left). Motion direction and strength are encoded by hue and saturation as indicated by the pseudo-color scales. Motion histograms (right) show proportional abundance of displacements’ angles weighted by their strength for the stimuli of the treadmill experiment [(A) to (C)] and for the cumulative motion signal of eight leaf displays and eight stripe displays from different cuttlefish individuals [(D) and (E)]. *R* indicates the weighted mean resultant vector alignment of each histogram (1 = fully aligned). *M* indicates the resultant motion strength. For the hunting sequences [(D) and (E)], *R* and *M* report averages and SD for the leaf and stripe displays.

Last, we used a custom-written biologically informed elementary motion detector (EMD) algorithm (*15*, *34*–*36*) to simulate the motion patterns perceived by a crab when looking at hunting broadclub cuttlefish. Videos were spatiotemporally downsampled to crab vision before computing their motion patterns. To validate the algorithm, we first tested it using the high-contrast expanding elliptical stimuli from the laboratory experiment (Fig. 4, A to C). The approach of an expanding gray ellipse produced a radially symmetrical motion pattern that is characteristic of approaching objects or predators (Fig. 4A) (*37*, *38*). The stimulus with static stripes instead produced a vertically bimodal motion pattern (Fig. 4B) that is induced by the horizontal dark edges of the stripes above and below the center of the approaching cue. Last, the moving stripe stimulus produced a downward unimodal motion pattern (Fig. 4C). The moving stripes stimulus produced 34 times more motion (as measured by resultant motion strength *M*) than the static stripes, while the motion generated by the gray disk without stripes was negligible in comparison. The gray disk without stripes had a weighted mean resultant vector alignment (*R*, a measure of directionality) of 0, as expected by its radially near-symmetrical expanding cue. The expanding stimulus with static stripes had a value of 0.09, whereas the moving stripes stimulus a value of 0.92. These results show that the expanding ellipse with moving stripes was effective at minimizing the radially expanding cue of the approaching stimulus.

To estimate the motion patterns elicited by hunting cuttlefish, we recorded, at 240 frames per second, additional hunting sequences in the wild from as close as possible to the viewpoint of a prey crab (Fig. 1, B to E). We filmed eight prey-view passing stripe hunting displays (movie S2) and eight “leaf” displays where the cuttlefish assumed the same body posture as the stripe display but turned pale olive green instead of passing dark stripes (movie S3) (*39*). We also estimated the motion patterns elicited by these leaf hunting sequences to allow a direct comparison between a hunting display with and without moving stripes. The leaf display produced a bimodal vertical motion pattern (Fig. 4D and movie S4), elicited by the body posture of the hunting cuttlefish with the wide arms extended sideways that create long and narrow horizontal approaching edges. In passing stripe hunting displays, the effects of this approaching cue are instead masked by the motion generated by the stripes, which generates a unimodal downward motion pattern (Fig. 4E and movie S5).

Across all the sequences filmed, the strength of the motion generated by the passing stripe displays was, on average, nine times stronger (as measured by resultant motion strength) than by the leaf displays. The mean weighted resultant vector alignment of the displays without stripes was 0.09 ± 0.06, which is substantially lower if compared to the 0.56 ± 0.08 value of the stripe displays. This highlights the fact that the leaf display still in part features a radially expanding component in its signal, whereas the passing stripe display does so to a much lesser extent due to the downward moving stripes. These results show that the passing stripe hunting display produces a motion pattern that is very different from that expected by approaching predators and that this is achieved by creating dynamic noise with the moving stripes.

## DISCUSSION

An approaching predator generates an expanding motion cue in the visual system of the prey (*37*, *38*). Given the importance of such information for survival, prey visual systems, particularly those of crabs, are finely tuned to detect expanding motion cues and respond to them with evasive action (*30*, *31*). For this reason, predators face a difficult challenge when attempting to conceal their approach. The results of this study provide evidence that broadclub cuttlefish use a previously undescribed strategy to camouflage their movements during the final moments of hunting crab prey. Instead of attempting to minimize the expanding motion cues associated with their approach, for example, by blending in with background levels of motion noise (*6*–*12*), the cuttlefish use the passing stripe display to generate strong downward motion that is not evolutionarily associated with predator approach. The weaker expanding motion cue of the hunting cuttlefish is overwhelmed by the stronger self-generated motion of the moving stripes. Here, we presented several strands of evidence supporting this mechanistic explanation for display function. First, we showed that an expanding stimulus overlayed with high-contrast downward moving stripes initiated fewer antipredator responses by crabs than stationary stripes or unstriped stimuli. While using crabs that are likely naïve to hunting predators displaying dynamic skin patterns was ideal to test the underlying mechanisms of this display, future studies could investigate whether natural prey items evolved any counteradaptations. Second, with a causal inference framework, we show that increasing the average speed of a cuttlefish approach results in higher temporal frequencies of stripes passing downward across the head, suggesting a strong causal link between the properties of the expanding motion signal and the downward moving motion noise. Third, expanding motion cues associated with approaching artificial and natural predator cues are altered by moving stripes in a simulated motion vision model. Together, these results suggest that the passing stripe display functions by overwhelming the threatening motion cues of the predator with nonthreatening downward movement of the stripes. This approach to camouflaging motion has not been described before in nature and represents a unique solution to a challenge faced during most visually guided predator-prey interactions.

When approaching prey with the passing stripe display (but also with the leaf display), the broadclub cuttlefish assumes a horizontally elongated body and arm posture, stretching the lateral arms out to the side and exposing only the flattened frontal body profile to the prey visual system. This posture in itself already reduces the strength of radially expanding motion cues: The motion field of the prey crab is dominated by upward and downward movement generated by the long horizontal cuttlefish outline. The lateral extension of the arms also has the added benefit of masking the mantle fin movements from the viewpoint of the crab, further minimizing the production of motion cues that could be linked to cuttlefish attack. It is suggestive to think that this dilution of the expanding horizontal motion field may also play a role in reducing the probability of adverse reaction by crab when cuttlefish predators approach slowly.

Other species of cephalopod are known to produce dynamic elements of body patterning in predator-prey interactions (*27*). Whether any of these patterns have evolved to perform similar motion camouflage functions remains to be demonstrated, so, for the moment, the broadclub cuttlefish is unique in its ability to camouflage its motion with moving stripes while stalking prey.

## MATERIALS AND METHODS

### Experimental animals

All procedures involving animals followed British and Indonesian laws and directives. Crab experiments in Bristol were conducted under the university investigation number (UIN) research permit 21/061. The observations in Indonesia were carried out under the research permits 63-64-65-66/SIP/IV/FR/2/2023 issued by the Indonesian National Research and Innovation Agency and the UIN permit 23-003 issued in Bristol. For the lab experiment, UK shore crabs *C. maenas* were collected by hand during low tide in rocky shores local to Bristol (51.44, −2.87), southwest England. Crabs were housed in individual containers in a shallow aquarium with fresh circulating seawater. Light conditions followed the natural cycle, salinity was kept at 30 parts per thousand, and all animals were released after 3 days. To film the hunting display of the broadclub cuttlefish in the field, Indonesian purple mangrove crabs *Metopograpsus frontalis* and mottled crabs *Grapsus albolineatus* were collected by hand along the coast and kept in a shallow water aquarium with fresh saltwater that was exchanged daily. The broadclub cuttlefish *S. latimanus* was filmed being presented with live prey in its natural environment.

### Treadmill experiment

UK shore crabs were tethered over a treadmill consisting of a Styrofoam ball (5 cm in radius) suspended on a flow of compressed air into a hemispherical holder (*40*). Crabs were tethered by gluing (with cyanoacrylate glue) a magnet to the dorsum of the crab, which was then connected to a 6-mm–diameter spherical ball bearing mounted on a horizontal rod. This allowed crabs to freely walk and rotate while maintaining their position in the center of the treadmill. A 24-inch (60.96 cm) 120-Hz gaming monitor (C24FG73FQU, Samsung, South Korea) was positioned against the only open side of a white fabric photo studio cube (50 cm by 50 cm by 50 cm) that surrounded the treadmill. The arena had a small opening at the top through which a digital camera (HC-X900M, Panasonic, Japan) recorded crab behavior. Stimuli were displayed on the monitor using a custom-written MATLAB script (R2022b, MathWorks, Natick, USA) that used the Psychophysics Toolbox extensions (*41*–*43*). Stimuli consisted of elliptical disks that expanded over a 10-s period following an exponential growth profile from a visual angle of 0° to a vertical angle of ~53° and a horizontal angle of ~74°, after which the stimuli remained fully expanded for another 5 s before disappearing. Stimuli consisted of a gray screen (negative control), a gray ellipse without stripes, and two striped (3 cycles of 1:5 ratio dark versus light gray stripes) ellipses with (4 Hz) or without (0 Hz) downward stripe motion. Each stimulus but the negative control was gradually increasing in contrast with the background, in 0.2 increments of WC, from 0.2 to 1. Contrasts were measured between the light stripe and the medium gray background of the monitor. The dark stripes were then added to the striped stimuli with a WC with the background of the same value but opposite sign. The radiance of each gray value of the monitor from 0 to 255 was measured using a spectrometer (Ocean HDX UV-VIS, Ocean Insight, Orlando, USA) coupled to an optic fiber (0.6 mm in diameter, 2 m in length; Ocean Insight). Radiances between 400 and 700 nm were used to compute the medium gray value of the monitor and the gray values to produce the elliptical stimuli of desired WC from crab visual perspective. The MATLAB script produced a continuous beep while the stimulus was expanding, which was used to synchronize crab videos with the expansion of the stimuli. The sound was directly input into the camera’s microphone via an audio cable so that it could not be heard on external speakers and potentially affect crab behavior. When placed on the treadmill, crabs were left to acclimatize with a medium gray screen for 1 min. The same gray screen was used in between stimulus presentations, which were started only if the crab was walking on the treadmill after at least a 30-s interval. When on the treadmill, crabs tended to walk undisturbed and then reacted suddenly when they first detected the expanding stimulus. We tested a total of 55 crabs, each subjected to all the stimuli in a randomized order. After the experiment, crabs were removed from the treadmill, the magnets were detached from their carapaces, and the crabs were returned to the aquarium. The presence or absence of behavioral response within the 10 s of stimulus expansion was scored manually, blinded to treatment. Behavioral responses included sudden freezing, sudden acceleration, tucking in claws and legs to assume a defensive posture, or extending the claws in an aggressive display (movie S6). The number of responses to the negative control stimulus (a zero-contrast expanding disc) across all crabs tested was used to calculate the probability of false positive responses.

### Stereo recordings of hunting cuttlefish

We recorded sequences of wild broadclub cuttlefish hunting with the stripe display in their natural environment from two different viewing angles using a stereo-camera rig. The rig comprised two action cameras (Hero 11, San Mateo, USA) filming at 240 frames per second (2.7K, wide, 16:9, stabilization features turned off) while mounted on each end of a 30-cm carbon fiber rod. To elicit the hunting display, mottled or purple mangrove crabs were presented to cuttlefish in the field by researchers using SCUBA. Each crab was tethered to a ~50-cm length of organic cotton thread on land and brought underwater housed in individual perforated plastic containers equipped with lids. Upon encounter with a cuttlefish, a crab was positioned and kept in place with the cotton thread on a transparent ~30-cm PVC disk in front of the camera rig until the cuttlefish detected the crab and started hunting. After the cuttlefish fed on the crab, we moved a 16.5 cm–by–10.5 cm laminated checkerboard (square size: 1.5 cm) in the field of view of the two cameras for calibration purposes. After the cuttlefish finished processing the prey, the thread, if still attached, was slowly pulled away from the cuttlefish and kept for disposal. Using custom written MATLAB routines with functions from the built-in MATLAB Stereo Camera Calibration Toolbox, each pair of videos was synchronized to the nearest frame by matching their audio tracks, and a series of calibration images featuring the checkerboard was used to calibrate the two GoPro cameras. This calibration process allowed lens distortion effects to be removed and to locate the relative position of the two cameras in the scene. We only kept calibrations with a <1 pixel mean reprojection error. Three-dimensional coordinates of cuttlefish and crab position were then determined from digitized pixel coordinates of a cuttlefish’s eye and a crab’s eye, which were semiautomatically tracked in each frame of the hunting sequence with a custom-written supervised template-matching system. From these coordinates, we reconstructed hunting trajectories and computed metrics such as starting distance of the hunting event and approach speed of the cuttlefish. When stripes were displayed, we also extracted the time interval between consecutive stripes, which was then converted to stripe frequency (hertz). Some individuals showed variation in stripe frequency within strikes, but this was substantially lower than the between-strikes variation (fig. S1). Similarly, we extracted the coordinates needed to calculate the length in centimeters of cuttlefish’s mantle, head, and feeding tentacles from selected frames of each hunting sequence. Cuttlefish sex was determined visually according to body patterning—males have a distinctive dark narrow line along the outer margin of the lateral fins (*22*, *44*). When the identity of individual cuttlefish was not immediately obvious, these were conservatively assigned using a combination of sex, location, and length of the fully extended tentacles.

### Motion detection algorithms

An EMD model was implemented on videos of artificial and natural predator cues using a custom-written script (R2022b, MathWorks, Natick, USA). The model is based on spatiotemporally correlated pixel intensity values quantified using an array of vertically and horizontally oriented EMDs. The first stage of the video processing removes all spatial and temporal contrasts that would not be visible to the receiver. Spatial contrast limits were based on spatial acuity limits of crabs (approximately 1 cycle/degree) (*29*, *45*–*47*), while temporal acuity was based on their critical flicker fusion frequency (cFFF; approximately 60 Hz) (*29*, *48*). To remove temporal information beyond the cFFF, we applied a temporal Gaussian blur with a σ value that caused the contrast of a stimulus flickering at the cFFF of 60 Hz to be reduced to 1% of its input amplitude. This spatio-temporally processed video is then passed through the EMD itself. The EMD uses horizontal and vertical detectors; the horizontal detector uses input from two pixels (*l* and *r* for left and right of two adjacent pixels, respectively) and across two consecutive frames (e.g., 0 and 1). The horizontal EMD contrast *h* is then calculated as

$$h=\left(l_{0}-l_{1}\right)\left(l_{0}-r_{1}\right)-\left(r_{0}-r_{1}\right)\left(r_{0}-l_{1}\right)$$

A gradient moving left will create a positive value, while a gradient moving right will create a negative value. The same principle is applied in the vertical plane. Last, this is converted into absolute values in the up, down, left, and right directions. This is a hypothetical motion detection model that—while well supported by behavioral evidence—is not designed to simulate specific neural pathways (*15*, *35*). Critically, by using video footage that has been recorded at framerates far beyond the cFFF and then smoothing the output to match receiver vision, our model controls for temporal aliasing artefacts that result from the progressive nature of video recordings. As such, our model does not generate “wagon-wheel” type motion artefacts that would not be visible to the receiver but is highly sensitive to “barber-pole” (aperture effect) motion.

The model was first applied to the videos of the high-contrast stimuli from the lab experiment. Then, we recorded new hunting sequences from as close as possible to the visual perspective of prey crab using one action camera (Hero 11, GoPro, USA) filming at 240 frames per second to estimate the motion patterns perceived by prey crab when looking at the “passing stripe” or an alternative “leaf” hunting display. The horizontal and vertical detector responses obtained from the videos were converted to strength and direction of displacements of each pixel between sequential pairs of video frames. From each video of the broadclub cuttlefish hunting (eight passing stripe, eight leaf displays), we only kept displacements that were greater than the 99th percentile of motion displacement of the background to remove unwanted noise. The obtained displacements were encoded using a color scale and summarized using motion histograms that display proportional abundance of displacements angles weighted by their strength (Fig. 4).

To calculate the resultant motion strength (*M*) of processed videos, we first calculated average horizontal and vertical detector responses for each of their frames. The sum of the two vector components of each frame was then used to calculate the resultant motion vector strength of each video. To calculate the weighted mean resultant vector alignment (*R*) of each video, we first calculated Cartesian coordinates for each pixel displacement for all the video frames. *R* was then calculated as the square root of the sum of the squared components of the vectors divided by their total strength. *R* ranges between 0 and 1 and is a proxy of the degree of vector alignment (1 indicates perfect alignment).

### Statistical analysis

All data analysis was conducted in R v4.3.2 (*49*) with the brms (*50*–*53*) package, which fits Bayesian models using Hamiltonian Monte Carlo via Stan (https://mc-stan.org/). Implemented routines followed the guidelines of McElreath (*33*). For all implemented models, we run four chains and obtained coefficient estimates from a total of 8000 postwarm-up samples. Models were run using within-chain parallelization implemented with the cmdstanr package. Visual inspection of trace plots, Monte Carlo SE, effective number of samples, and R-hat values indicated model convergence. Models were further assessed using posterior predictive model checking, which compares model predictions with observed data.

#### Effect of passing stripe display on prey response

To investigate the effects of overlaying moving stripes on expanding predator stimuli on the response to expanding stimuli by crabs, we modeled the probability of crab response using a Bernoulli distribution with logit-link. The model included the main predictor stimulus (gray disk, static stripes disk, and moving stripes disk) and a Gaussian process term contrast (WC with the background, from 0.2 to 1) grouped by stimulus. We further included the random intercept term crab_ID (*N* = 55) to account for the repeated observations of each crab and the random slopes over stimulus and contrast because their relationship with the probability of response varied among crabs. This model was implemented using weakly informative prior distributions [normal with mean = 0 and SD = 1.5 for the coefficients of each stimulus, exponential (1) for the sdgp parameter of the Gaussian process and for SDs of the multilevel hyperparameters]. For graphical display, we present median response probabilities and their 95% CIs of the posterior distribution of fitted values for the population average for each stimulus as a function of WC with the background (Fig. 2A). We present results as comparisons (median differences and their 95% CIs) between response probabilities predicted by the model for each pair of stimuli along the WB range (Fig. 2B).

#### Stereo recordings of hunting cuttlefish

The DAG of Fig. 3B shows the causal relationships between the variables that are believed to influence the frequency of the stripes. Please note that this graph does not only show measured variables but also unobserved ones such as strength of current, water turbidity, or presence of cuttlefish predators. These are believed to influence the approach speed of the cuttlefish. We focused on investigating the direct (and total) effect of cuttlefish’s approach speed on stripe temporal frequency. To describe this effect, we included in the model approach speed in centimeters per second as a main predictor of stripe frequency in hertz, but we also added the covariates crab carapace width and length of cuttlefish head in centimeters, as these are confounders of the causal path of approach speed and should be controlled for, and the categorical predictor sex of the cuttlefish (female or male), which is not a confounder, but rather a concurrent cause of stripe frequency and adding it to the model minimizes potential bias. This model formulation makes all the included predictors conditionally independent, which allows estimating the direct causal effects of cuttlefish approach speed, sex, size, and crab size on stripe frequency. All numerical variables were standardized to mean = 0 and SD = 1, and we modeled temporal stripe frequency in hertz using a Student *t* distribution with ν = 2 to allow for thicker tails and reduce the influence of extreme values. We further included the random intercept term cuttlefish_ID (*N* = 17) to account for the repeated observations of each cuttlefish. This model was implemented using weakly informative prior distributions [normal with mean = 0 and SD = 0.5 for the coefficients of cuttlefish sex, normal with mean = 0 and SD = 1 for the rest of the coefficients, exponential (1) for the SDs of the intercepts and for the σ parameter of the Student *t* distribution]. We used data simulation to validate the model by testing that it could recover the simulated parameters. For displaying the results, we report median stripe temporal frequencies and their 95% CIs of the posterior distribution of fitted values for the population average when (i) increasing approach speed of one SD, for an average size male hunting an average size crab prey, (ii) increasing width of crab’s carapace of one SD, for an average size male hunting at average approach speed, (iii) increasing length of cuttlefish’s head of one SD, for a male hunting an averaged sized crab at average approach speed, and (iv) changing sex of an average sized cuttlefish hunting an average sized crab at an average approach speed.
