# Supplementary material for: Stealth and deception: Adaptive motion camouflage in hunting broadclub cuttlefish
Source: Sci Adv. 2025 Mar 26;11(13):eadr3686. doi: 10.1126/sciadv.adr3686 (PMC11939058; doi:10.1126/sciadv.adr3686)
Supplement: Supplementary file 1 — Fig. S1 Tables S1 and S2 Legends for movies S1 to S6 [file sciadv.adr3686_sm.pdf]

Science Advances
AAAS

Supplementary Materials for

Stealth and deception: Adaptive motion camouflage in hunting broadclub cuttlefish

Matteo Santon *et al.*

Corresponding author: Matteo Santon, matteo.santon@bristol.ac.uk

Sci. Adv. **11**, eadr3686 (2025)
DOI: 10.1126/sciadv.adr3686

The PDF file includes:

Fig. S1
Tables S1 and S2
Legends for movies S1 to S6

Other Supplementary Material for this manuscript includes the following:

Movies S1 to S6

Fig. S1.

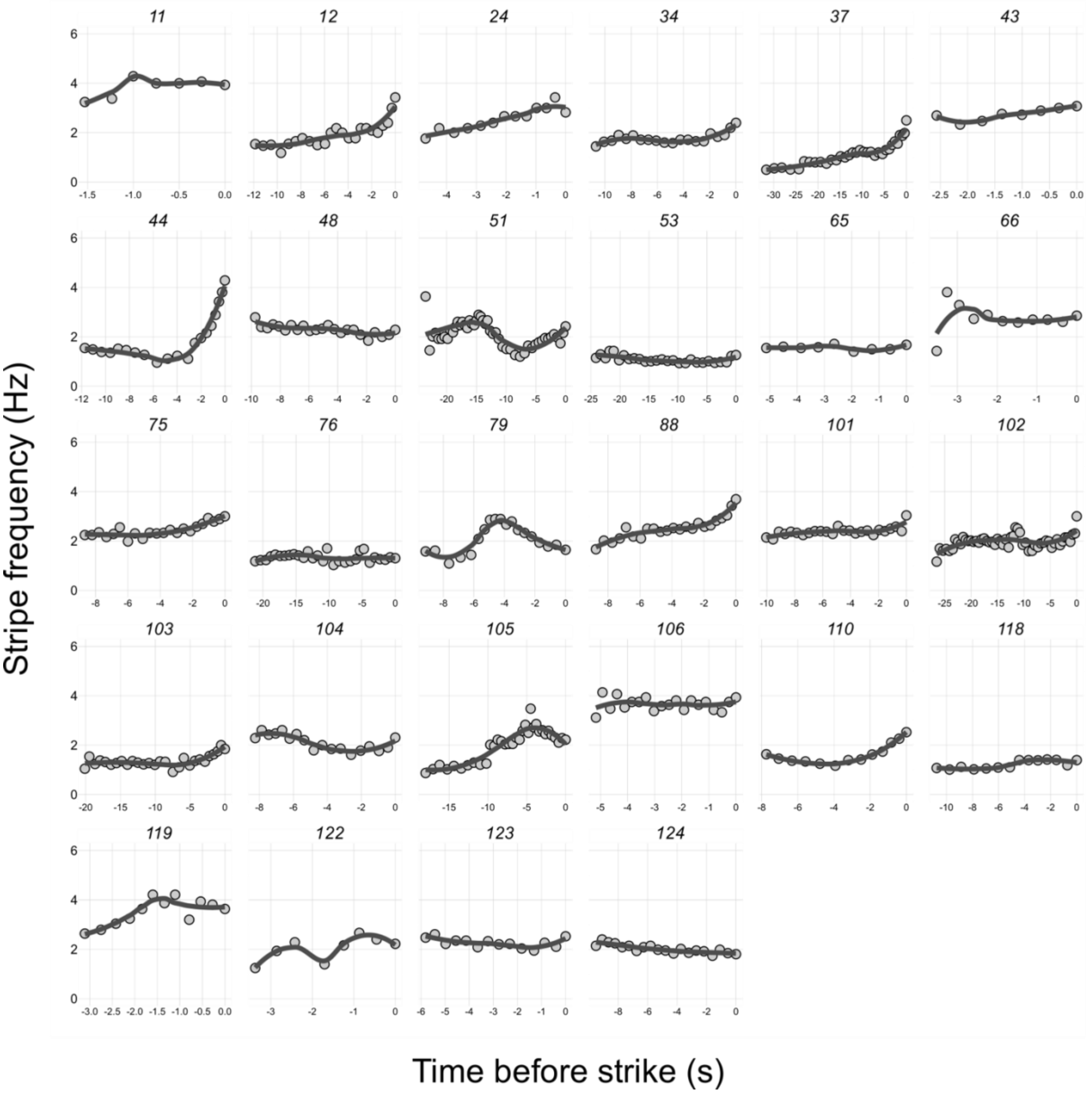

Stripe temporal frequency does not vary substantially within strikes. Filled circles show the stripe temporal frequency of each stripe passed by cuttlefish as a function of the time before the strike. Solid lines represent smooth curves that show the general trends. Each numbered panel indicates a different strike.

Table S1.

**Parameters**	**Mean**	**M. Error**	**95 % CI**	
			**Low**	**High**
**Regression coefficients**				
Grey disk	0.05	0.89	-1.78	1.77
Static stripes disk	-0.35	1.05	-2.45	1.75
Moving stripes disk	-0.69	1.07	-2.74	1.47
**Gaussian Process Hyperparameters**				
Sdgp [Contrast, Grey disk]	1.46	0.66	0.61	3.10
Sdgp [Contrast, Static stripes disk]	1.99	0.78	0.94	3.88
Sdgp [Contrast, Moving stripes disk]	2.26	1.01	0.83	4.70
Lscale [Contrast, Grey disk]	0.50	0.13	0.29	0.80
Lscale [Contrast, Static stripes disk]	0.48	0.11	0.29	0.72
Lscale [Contrast, Moving stripes disk]	0.42	0.09	0.27	0.64
**Multilevel Hyperparameters for Crab_ID (N = 55)**				
Sd Grey disk [Intercept]	0.81	0.23	0.37	1.28
Sd Static stripes disk	0.49	0.34	0.02	1.25
Sd Moving stripes disk	0.71	0.35	0.06	1.38
Sd Contrast	0.33	0.27	0.01	0.99
Cor [Intercept, Static stripes disk]	0.06	0.35	-0.62	0.70
Cor [Intercept, Moving stripes disk]	-0.43	0.31	-0.86	0.35
Cor [Static stripes disk, Moving stripes disk]	-0.12	0.36	-0.75	0.60
Cor [Intercept, Contrast]	-0.11	0.39	-0.77	0.65
Cor [Static stripes disk, Contrast]	-0.05	0.38	-0.74	0.68
Cor [Moving stripes disk, Contrast]	-0.02	0.38	-0.71	0.70

Effect of passing stripe display on prey response. Parameter estimates for model investigating the likelihood of crabs responding to the expanding stimuli as a function of stimulus type and Weber Contrast with the background.

Table S2.

**Parameters**	**Mean**	**M. Error**	**95 % CI**	
			**Low**	**High**
**Regression coefficients**				
Female	0.13	0.29	-0.43	0.72
Male	-0.08	0.22	-0.51	0.36
Cuttle head length	-0.03	0.22	-0.47	0.40
Approach speed	0.71	0.15	0.44	1.03
Crab carapace width	0.15	0.12	-0.10	0.37
**Multilevel Hyperparameters for Cuttlefish ID (N = 17)**				
Sd [Intercept]	0.47	0.16	0.15	0.81
**Distributional parameters**				
Sigma	0.29	0.09	0.15	0.50
Nu	2	0	2	2

Cuttlefish approach speed influences stripe temporal frequency. Parameter estimates for model investigating cuttlefish stripe temporal frequency as a function of cuttlefish's approach speed, sex, size, and crab prey size. All numeric predictors were standardized to mean = 0 and S.D. = 1.

Movie S1. Synchronised left and right camera view of a broadclub cuttlefish hunting with the passing stripe display.

Movie S2 Prey crab view of a broadclub cuttlefish hunting with the passing stripe display.

Movie S3. Prey crab view of a broadclub cuttlefish hunting with the leaf display.

Movie S4. EMD model output for Movie S3. Strength and direction of displacements perceived by prey crab are encoded using the false colour scale shown in Fig.4.

Movie S5. EMD model output for Movie S2. Strength and direction of displacements perceived by prey crab are encoded using the false colour scale shown in Fig.4.

Movie S6. Example video of crab responses to the expanding stimuli used in the treadmill experiment.
