# Supplementary material for: Stealth and deception: Adaptive motion camouflage in hunting broadclub cuttlefish
Source: Sci Adv. 2025 Mar 26;11(13):eadr3686. doi: 10.1126/sciadv.adr3686 (PMC11939058; doi:10.1126/sciadv.adr3686)
Supplement: Supplementary file 2 — Movies S1 to S6 [file sciadv.adr3686_movies_s1_to_s6.zip › sciadv.adr3686.pdf]

Science Advances
AAAS

Supplementary Materials for

Stealth and deception: Adaptive motion camouflage in hunting broadclub cuttlefish

Matteo Santon *et al.*

Corresponding author: Matteo Santon, matteo.santon@bristol.ac.uk

Sci. Adv. **11**, eadr3686 (2025)
DOI: 10.1126/sciadv.adr3686

The PDF file includes:

Fig. S1
Tables S1 and S2
Legends for movies S1 to S6

Other Supplementary Material for this manuscript includes the following:

Movies S1 to S6
